# MRPS16 promotes lung adenocarcinoma growth via the PI3K/AKT/Frataxin signalling axis

**DOI:** 10.1111/jcmm.18166

**Published:** 2024-03-20

**Authors:** Zaixing Cheng, Kaming Xue, Cui Xiong, Zhikun Zheng, Jinsong Li, Xinwei Qiao

**Affiliations:** ^1^ Department of Thoracic Surgery Union Hospital, Tongji Medical College, Huazhong University of Science and Technology Wuhan Hubei China; ^2^ Department of Traditional Chinese Medicine Union Hospital, Tongji Medical College, Huazhong University of Science and Technology Wuhan Hubei China; ^3^ Department of Endocrinology Union Hospital, Tongji Medical College, Huazhong University of Science and Technology Wuhan Hubei China

**Keywords:** lung adenocarcinoma, MRPS16, PI3K/AKT/Frataxin, proliferation

## Abstract

Although MRPS16 is involved in cancer development, its mechanisms in developing LAUD remain unclear. Herein, qRT‐PCR, WB and IHC were utilized for evaluating MRPS16 expression levels, while functional assays besides animal experiments were performed to measure MRPS16 effect on LAUD progression. Using WB, the MRPS16 effect on PI3K/AKT/Frataxin signalling pathway was tested. According to our study, MRPS16 was upregulated in LAUD and was correlated to the advanced TNM stage as well as poor clinical outcomes, which represent an independent prognostic factor. Based on functional assays, MRPS16 is involved in promoting LAUD growth, migration and invasion, which was validated further in subsequent analyses through PI3K/AKT/Frataxin pathway activation. Moreover, MRPS16‐knockdown‐mediated Frataxin overexpression was shown to restore the reduction in tumour cells proliferation, migration and invasion. Our results revealed that MRPS16 caused an aggressive phenotype to LAUD and was a poor prognosticator; thus, targeting MRPS16 may be effectual in LAUD treatment.

## 1 INTRODUCTION

Based on the latest global cancer statistics report, most cancer deaths are attributable to lung cancer, the most prevalent malignancy.^1^ In this study, 80% of patients with non‐small‐cell lung cancer (NSCLC), accounting for about 83% of primary lung cancer, are diagnosed at an advanced stage because of lacking early detection biomarkers, and lung adenocarcinoma (LAUD) accounts for the vast majority of NSCLC.^2^ The advanced LAUD patient prognosis is poor; hence, in order to diagnose and effectively treat LAUD early, it is crucial to seek potential biomarkers besides developing therapeutic targets.

To identify candidates worth further investigation, high‐throughput proteomics was used between paired normal lung tissue and LAUD, revealing that MRPS16 was overexpressed in LAUD more than in normal paired lung tissue. The 137 amino acid protein encoded by the MRPS16 gene is the most conservative mitochondrial ribosomal protein from mammals to yeast. Approximately 40% of sequences of the human protein are similar to that of bacteria.^3^ There are two subfamilies of mitochondrial ribosomes in humans: SSU and LSU.^4^ The essential step in the SSU assembly is when ribosomal protein S16 binds to a small crevice.^5^ It is necessary for extraribosomal enzymes to introduce nicks into supercoiled DNA molecules.^6^ An early stop codon present in MRPS16 will result in an unstable, truncated protein.^7^ A sharp decline in 12S rRNA levels indicates that the mutation is harmful.^8^ The mutation in Drosophila's mitochondrial ribosomal protein 12S disrupted the protein assembly in the active ribosome, causing a reduction in 12S rRNA levels.^9^, ^10^ Hong Zhang et al^11^ reported that the dysregulation of mitochondrial ribosomal proteins and their corresponding genes in relation to cellular apoptosis and pathological conditions. Linlin Liu et al^12^ reported that the bioinformatics results provide evidence of the potential of mitochondrial ribosomal genes as cancer biomarkers. Annamaria Gal et al^13^ and Wei Zhang et al^14^ reported the strong correlation between mitochondrial translation genes and NSCLC prognosis. Wang et al^15^ revealed that MRPS16 enhances tumour cell growth by PI3K/AKT signal path, demonstrating the critical role played by MRPS16 in biological processes further. The MRPS16 roles, especially in LAUD, remain unexplained to a great extent.

Herein, both loss and gain of the functional experiments in vitro and in vivo showed that MRPS16 enhances LAUD cell growth, migration and invasion. Moreover, Cignal Finder Cancer 10‐Pathway Reporter Kits were used for screening the potential signalling pathways participating in this process. Eventually, PI3K/AKT signalling axis was found to be significantly suppressed by MRPS16 knocking down in H23 and H2030 cells, in contrast to the other signalling axis. Furthermore, based on GSEA, MRPS16 showed a significant correlation to the PI3K/AKT signalling pathway. Moreover, rescue experiments were conducted to validate the above finding further. Collectively, PI3K/AKT/Frataxin signalling axis activation by MRPS16 was indicated to enhance tumour growth.

## 2 MATERIALS AND METHODS

### 2.1 Reagents and cell lines

Normal lung epithelium MEL12 and NL20, multiple LAUD cell lines H1755, H23, H2030 and H1734 were cultivated according to the supplier's cultivation suggestion, with more available details in Appendix S1.

### 2.2 LAUD samples

Between September 2017 and June 2021, 98 LAUD samples and corresponding paracancerous paired samples were acquired from Wuhan Union Hospital, with the informed consent of participants as well as the approval of relevant research ethics committees. Herein, no patients had adjuvant, neoadjuvant or radiotherapy prior to the surgery besides collecting and managing the clinical samples and data according to the appropriate guidelines and regulations. For more detailed details, see Tables S1 and S2.

### 2.3 Plasmid construction and lentivirus

Constructing the overexpression plasmid, pLVX‐Puro‐MRPS16, was followed by treating H23 and H2030 cells with the corresponding expression plasmid, listing the precise procedure in Appendix S1. Briefly, after growing H23 and H2030 cells to 90% confluency in six‐well plates, they were transfected following the protocols. After being infected for 48 h, puromycin was used to select the cells for 5–6 days for the purpose of removing uninfected cells. Based on the MRPS16 sequence, siRNAs were designed, selecting the two siRNAs having the most significant knockout impact. For more detailed details, see Appendix S1.

### 2.4 CCK‐8 assay

Using CCK‐8 (Cell Counting Kit‐8, Dojindo, Tokyo, Japan), a cell proliferation assay took place following the protocols. After seeding the cells into a 96‐well plate (5 × 10^5^ cells/well) using an appropriate fresh medium and treating them with CCK‐8 solution, cell proliferation was determined treatment, measuring the absorption by a microplate reader (Bio‐Rad Laboratories, Hercules, CA, USA), with more available details in the previous publication.^16^, ^17^, ^18^

### 2.5 Western blotting (WB)

Using both RIPA buffer and BCA (beyotime, Shanghai, China), tissues and cells were lysed and quantified, respectively. After subjecting the lysates to SDS–PAGE, they were transferred to PVDF, which was followed by incubation with corresponding antibodies under the appropriate conditions, and then was succeeded by subjecting to a colour reaction. Primary antibody information is referred to in ‘Cell lines and reagents’, with more available details in the previous publication.^19^, ^20^, ^21^

### 2.6 Quantitative real‐time PCR (qRT‐PCR)

Performing total RNA extraction, cDNA synthesis and qRT‐PCR took place, as mentioned earlier. The calculation of relative mRNA expression levels was relative to β‐actin, with more available details in Appendix S1.

### 2.7 Colony formation assay

A total of 400 cells were resuspended and cultured for 2 weeks into an unchanged complete culture medium, describing the detailed procedures in Appendix S1.

### 2.8 Cignal finder cancer 10‐pathway reporter array

Briefly, we transfected common cancer pathways with luciferase reporters on the resuspended cells in 96‐well plates. Following the incubation, the cells were tested for luciferase activity following the protocols, with more available details in Appendix S1.

### 2.9 EdU proliferation assay

EdU fluorescence staining was utilized to evaluate newly synthesized DNA after the indicated H23 and H2030 cell treatments following the protocols. In brief, cell seeding took place in 96‐well plates at an appropriate density, subsequently treated with EdU, followed by formalin fixation. Then wash three times with PBS and use Triton X‐100 for cell permeabilization. Finally, cells followed treatment with the corresponding luminescent reagent, with more available details in Appendix S1.

### 2.10 Immunohistochemistry (IHC) and Immunofluorescence (IF)

IHC and IF details are available in our previously published articles.^22^ In brief, after dehydrating, paraffin‐embedding and sectioning the tissues, they were incubated using appropriate primary and secondary antibodies.

### 2.11 Tumour xenograft model

From Beijing Vital River Animal Technology Co. Ltd. (Beijing, China), 40 nude mice were purchased. Tongji Medical College's Animal Experiments Committee approved all animal studies. A humane endpoint was established in our study following AAALAC guidelines, with more available details in Appendix S1.

### 2.12 Statistical analysis

Utilizing SPSS 23.0 software (SPSS, Inc., Chicago, IL, USA), R 4.0.2 software (http://www.r‐project.org/) as well as GraphPad Prism (version 8.0; GraphPad Inc., La Jolla, CA, USA), the statistical analyses were performed, reporting the data as mean ± SD. Moreover, using unpaired/paired Student's *t* test for two groups or one‐way anova + Dunnett's for more than two groups for assessing statistically significant data. The chi‐square test, Pearson's correlation and one‐way analysis of variance were also conducted. Cox regression analysis and log‐rank test were utilized for determining survival difference and hazard ratio. P < 0.05 indicated statistically significant.

## 3 RESULTS

### 3.1 MRPS16 was overexpressed in LAUD and negatively correlated to prognosis

**FIGURE 1:**
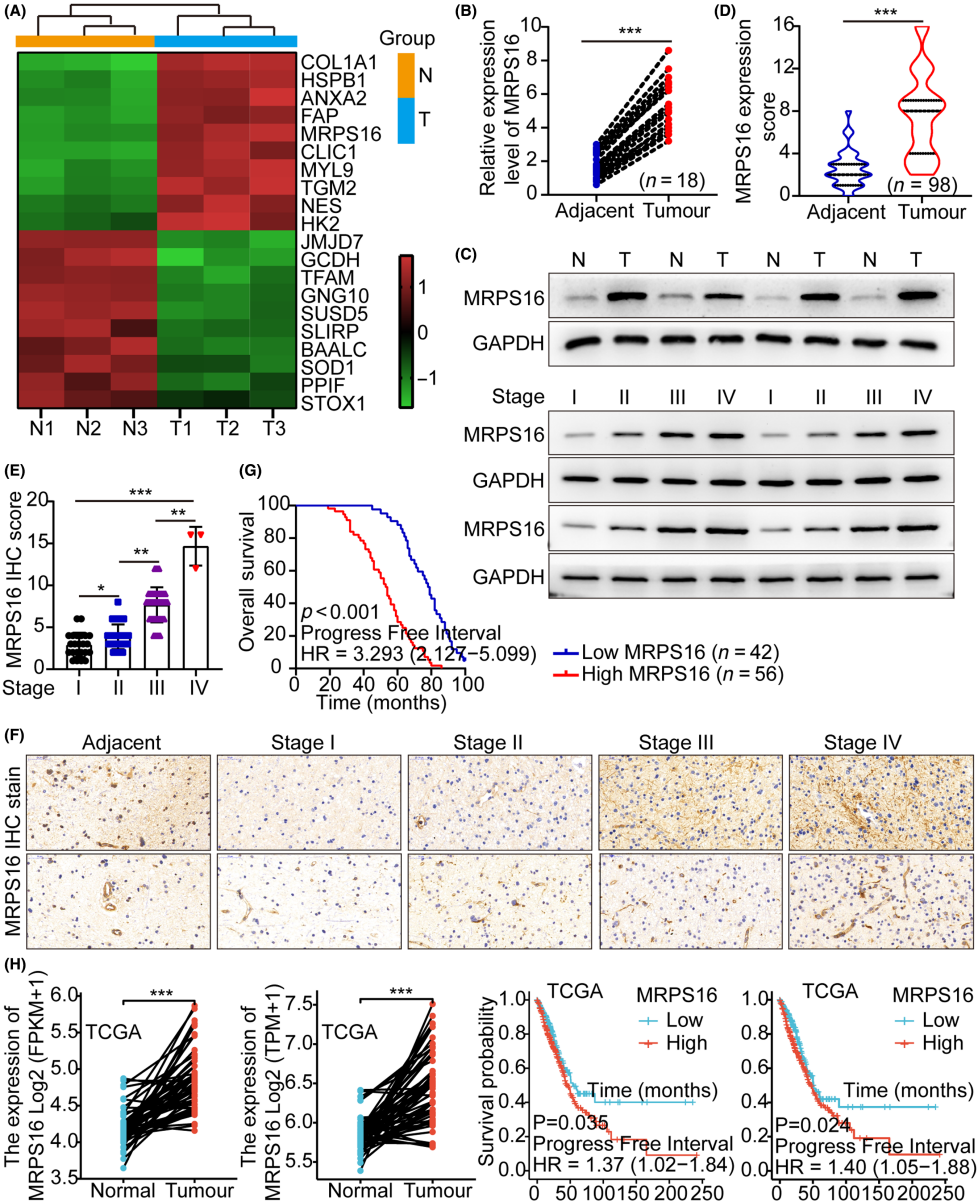
MRPS16 was overexpressed in lung adenocarcinoma and negatively associated with prognosis. (A) Heatmap shows the top up/downregulated genes in different groups. (B) qRT‐PCR was used to detect MRPS16 mRNA levels in lung adenocarcinoma tissues and adjacent tissues. (C) WB was used to detect the expression of MRPS16 protein in lung adenocarcinoma tissues and paired normal lung tissue and tumour tissues of different grades (I–IV). (D–F) IHC staining and scoring were used to detect the protein levels of MRPS16 in lung adenocarcinoma tissues and adjacent tissues and tumour tissues of different grades (I–IV). (G) Log‐rank test of OS was conducted. (H) The results of TCGA database showed that MRPS16 was highly expressed in tumour tissues, and the expression level was inversely proportional to the prognosis of patients. Data were presented as mean ± SD from three independent experiments. *p < 0.05, **p < 0.01 and ***p < 0.001.

For identifying the candidate genes contributing to tumour progression, high‐throughput sequencing was used (T vs. N, T: LAUD; N: paired normal lung tissue). The top 10 upregulated and downregulated genes were altered more than fivefold in LAUD compared to paired normal lung tissue (Figure 1A), among them, MRPS16 showed a significant overexpression. Based on WB and qRT‐PCR, MRPS16 was significantly overexpressed in tumour tissues and increased with tumour grade more than in adjacent tissue and paired normal lung tissue (Figure 1B,C) and based on IHC, the typical IHC staining micrographs in Figure 1G reveal that MRPS16 IHC staining intensity showed a significant enhancement in tumour tissues and was increased with tumour grade more than in adjacent tissue. Additionally, IHC staining score was positively correlated to malignancy grade (Figure 1D,E). Based on Kaplan–Meier analysis, patients with MRPS16 overexpression had a poorer prognosis (Figure 1F,H). Interestingly, data analysis from the TCGA tumour database also showed again that MRPS16 was overexpressed in tumour tissues and was inversely proportional to the patient prognosis (Figure 1H and Figure S1A). Here we used control samples and tumour samples from TCGA. Considering that control samples in TCGA most often suffer from uneven sample distribution, this seems to be an inevitable shortcoming, which may amplify the detection of differences and emphasize clinically irrelevant statistical differences. Therefore, although statistical significance can be determined, clinical significance may be compromised.

Performing the ROC analysis between MRPS16‐based, TNM‐based and a combination of both for assessing MRPS16 pathological and clinical predictive value to predict the clinical outcomes revealed that, based on AUC, the combination model (0.758) could predict clinical outcomes better than the TNM‐based model alone (0.652) (Figure S1B,C). Moreover, the examined association between MRPS16 mRNA levels and clinicopathological characteristics in 98 LAUD samples (Table S1) showed that the MRPS16 mRNA expression level had a significant correlation to the tumour size (*p* = 0.002), lymph node metastasis (*p* < 0.0001) and TNM stage (*p* = 0.002). Based on univariate and multivariate Cox regression analyses, MRPS16 mRNA overexpression was correlated to TNM stage and lymph node metastasis; hence, it was an independent prognostic factor for poor survival of LAUD patients (Table S2), indicating that MRPS16 might be a potential LAUD biomarker.

### 3.2 MRPS16 overexpression facilitates tumour cell growth

**FIGURE 2:**
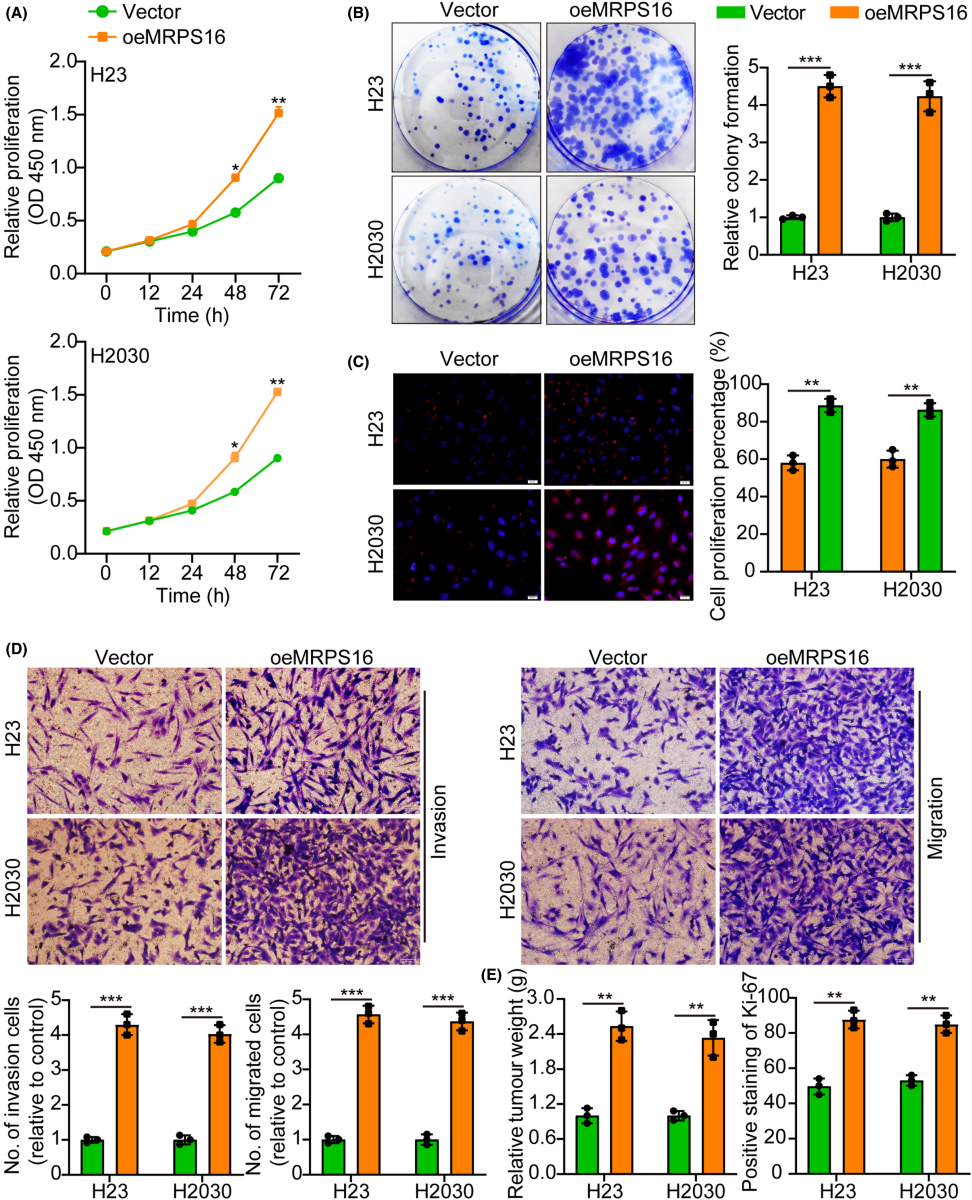
Overexpression of MRPS16 facilitates tumour cell growth. (A) Cell growth curves measured by CCK‐8 between Vector and oeMRPS16. (B) MRPS16 overexpression facilitated colony formation and histogram quantification (panels). (C) Cell growth curves measured by EdU between Vector and oeMRPS16. The left side represents the histogram. (D) Transwell migration and invasion assays show that overexpression of MRPS16 facilitates cell migration and invasion. The numbers of migrants and invading cells are shown. Bars: 50 μm. (E) Representative histogram of tumour weight and Ki‐67 staining between Vector and oeRNF7. Data were presented as mean ± SD from three independent experiments. *p < 0.05, **p < 0.01 and ***p < 0.001.

The first step was to measure MRPS16 protein in two normal lung epithelial cells (MEL12 and NL20) and four LAUD cell lines (H1755, H23, H2030 and H1734) by WB. According to the results, MRPS16 protein levels are higher in the LAUD cell line than in normal lung epithelium (Figure S1D). Therefore, we selected two cell lines, H23 and H2030, with moderate expression of MRPS16 protein for subsequent research. Next, overexpressed MRPS16 in H23 and H2030 cells were verified using WB (Figure S1E). Based on functional tests, MRPS16 overexpression advanced tumour cell proliferation, migration and invasion (Figure 2A–D). Furthermore, in vivo animal experiments once again showed that overexpression of MRPS16 promotes tumour growth. Ki‐67 IF staining was utilized for assessing the proliferative tumour index, indicating that proliferation in the MRPS16 overexpression groups was more elevated than in the control groups (Figure 2E and Figure S2A), suggesting that MRPS16 overexpression enhances tumour growth.

### 3.3 MRPS16 knockdown suppresses tumour cell growth

**FIGURE 3:**
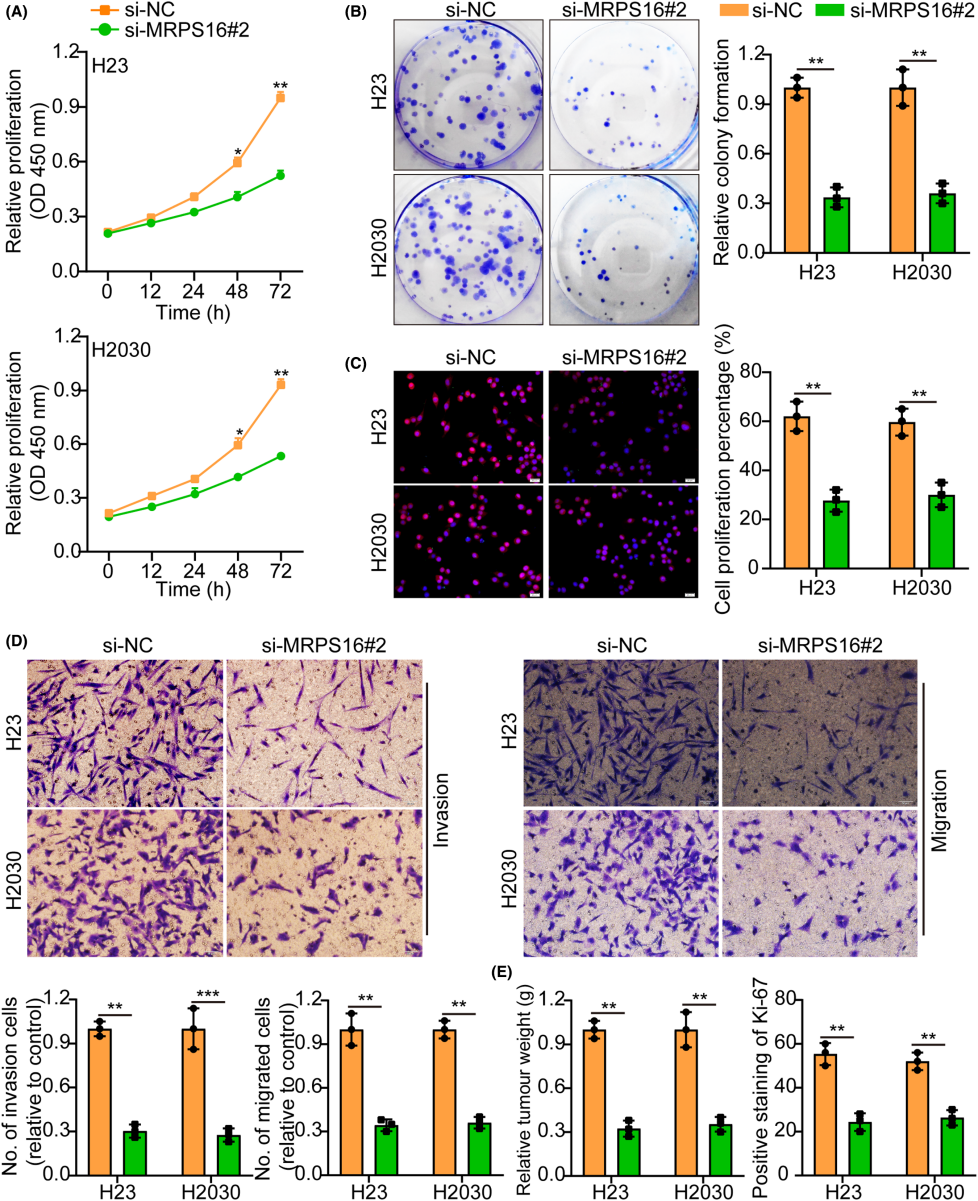
Knockdown of MRPS16 suppresses tumour cell growth. (A) Cell growth curves measured by CCK‐8 assay between si‐NC and si‐MRPS16#2. (B) MRPS16 knockdown inhibited colony formation and histogram quantification (panels). (C) Cell growth curves measured by EdU between si‐NC and si‐MRPS16#2. The left side represents the histogram. (D) Transwell migration and invasion assays show that the knockdown of MRPS16 inhibited cell migration and invasion. The numbers of migrants and invading cells are shown. Bars: 50 μm. (E) Representative histogram of tumour weight and Ki‐67 staining between si‐NC and si‐MRPS16#2. Data were presented as mean ± SD from three independent experiments. *p < 0.05 and **p < 0.01.

MRPS16 knocked down efficiency in H23 and H2030 cells were validated by WB (Figure S1F). As the si‐MRPS16#2 with the highest knockdown efficiency, it will be used as a follow‐up study. Functional tests indicated that MRPS16 knocking down curbed tumour cells proliferation, migration and invasion (Figure 3A–D), which was verified further by the in vivo animal experiments. Performing Ki‐67 IF staining for the purpose of assessing, the proliferative tumour index showed that proliferation in the MRPS16 knockdown groups was reduced more than in the control groups (Figure 3E and Figure S2B). These data suggest that MRPS16 knockdown inhibits tumour growth.

### 3.4 MRPS16 activates the PI3K/AKT signalling

**FIGURE 4:**
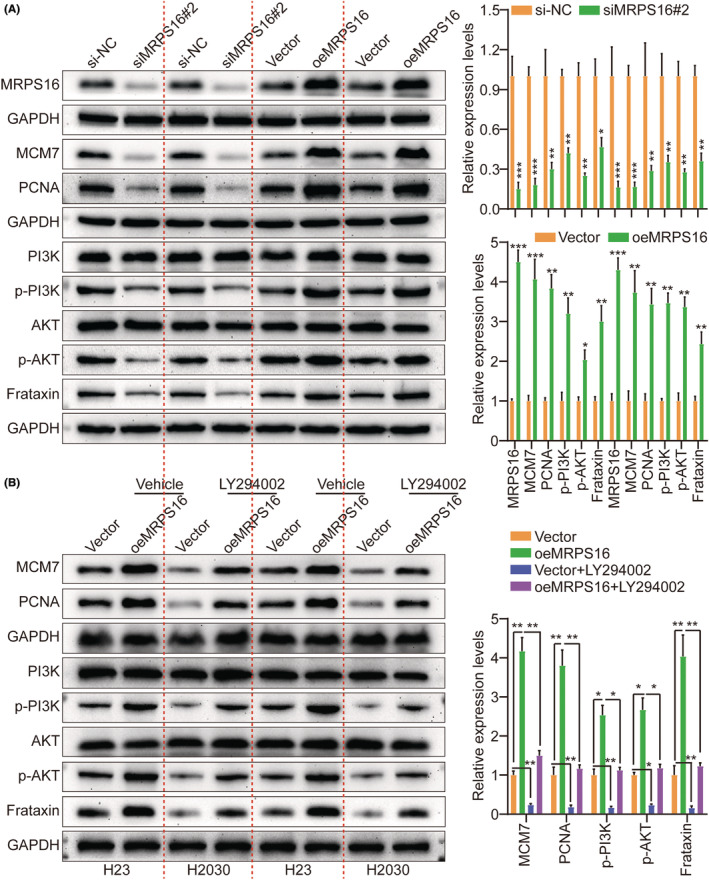
MRPS16 activates the PI3K/AKT signalling. (A) Expressions of MRPS16, MCM7, PCNA, AKT, p‐AKT, PI3K, p‐PI3K and Frataxin were detected by WB between si‐NC, siRNF7#2, Vector and oeMRPS16. The left side represents the histogram. (B) Expression of MCM7, PCNA, AKT, p‐AKT, PI3K, p‐PI3K and Frataxin were detected by WB between Vector, oeMRPS16, Vector+LY294002 and oeMRPS16+LY294002. The left side represents the histogram. Data were presented as mean ± SD from three independent experiments. *p < 0.05 and **p < 0.01.

Based on Cignal finder cancer 10‐pathway reporter array used for screening the potentially involved signalling pathways in this process, PI3K/AKT signalling axis was significantly inhibited by MRPS16 knocking down in H23 and H2030 cells, in contrast to the other signalling axis (Figure S3A). Moreover, GSEA revealed that MRPS16 was significantly correlated to PI3K/AKT signalling (Figure S3B). Additionally, performing MRPS16 knocking down and overexpressing in H23 and H2030 cells, respectively, for the purpose of validating the ability of MRPS16 to promote tumour growth further through PI3K/AKT pathway activation indicated that PI3K (p‐PI3K) and AKT (p‐AKT) phosphorylation levels, not the total PI3K and AKT, besides MCM7 and PCNA proliferation‐related proteins were downregulated by MRPS16 knocking down while promoted by MRPS16 overexpression (Figure 4A), indicating that MRPS16 activates the PI3K/AKT pathway.

### 3.5 MRPS16 influences tumour growth through PI3K/AKT signalling activation

**FIGURE 5:**
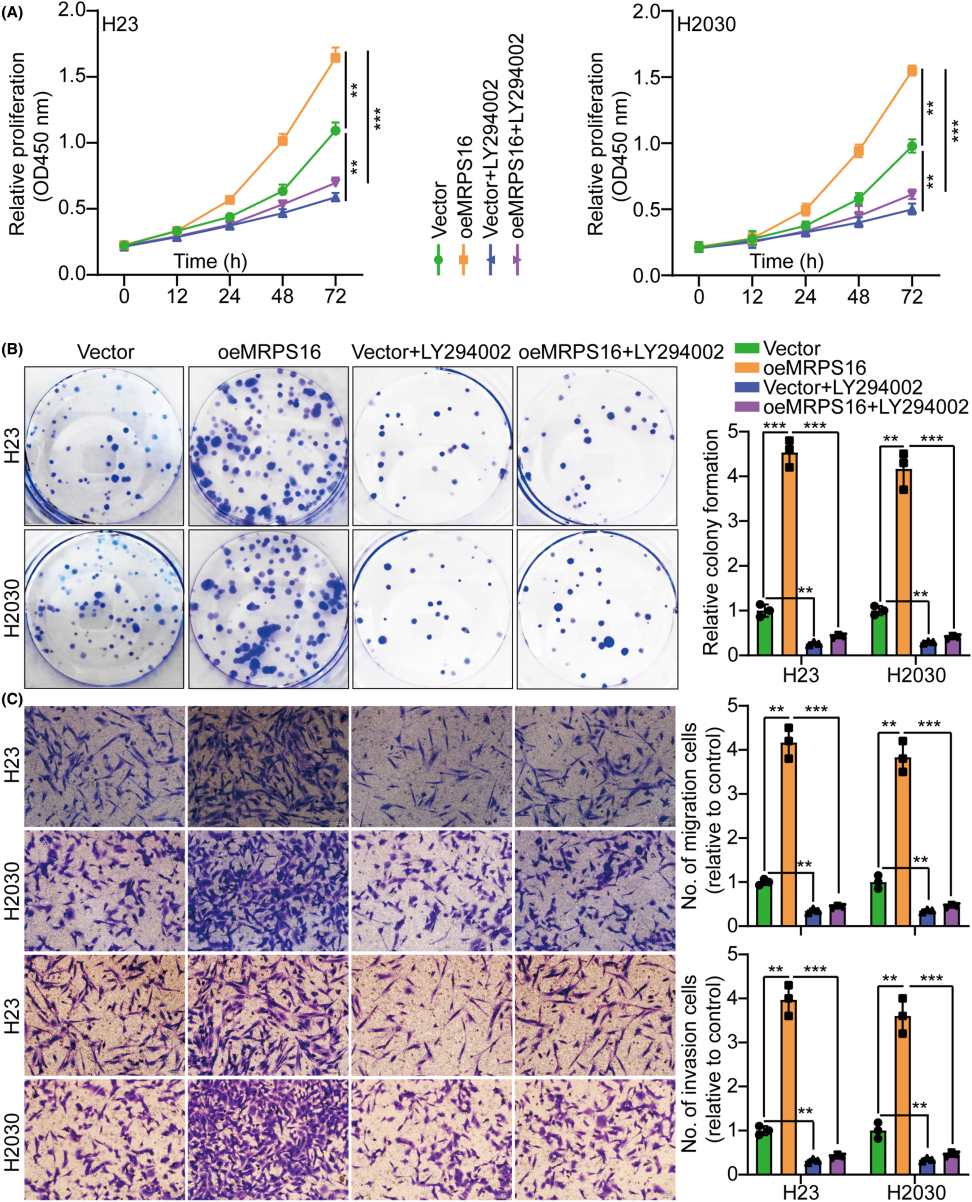
MRPS16 influences tumour growth by activating this PI3K/AKT signalling. (A) Cell growth curves measured by CCK‐8 assay in different treatment groups. (B) Cell growth was measured by colony formation assay in different treatment groups and histogram quantification (panels). (C) Transwell migration and invasion assays in different treatment groups and histogram quantification (panels). Data were presented as mean ± SD from three independent experiments. **p < 0.01 and ***p < 0.001.

By not only treating H23 and H2030 cells with PI3K/AKT inhibitor LY294002 to further determine the MRPS16 ability to promote cell proliferation through PI3K/AKT activation but also by investigating MRPS16 overexpression effect on proliferation, it was revealed that consistent with the earlier conclusion, MRPS16‐OE induced p‐PI3K and p‐AKT but not PI3K and AKT, as well as upregulated MCM7 and PCNA. Nevertheless, this effect was weakened on treating H23 and H2030 cells with PI3K/AKT inhibitor LY294002 (Figure 4B). Additionally, based on the CCK‐8, colony formation, Transwell migration/invasion and EdU assays, LY294002 led to a significant reverse of the MRPS16 effect on enhancing H23 and H2030 cell proliferation (Figure 5A–C and Figure S3C), which was parallel to the in vitro study which verified that LY294002 treatment significantly decreased tumour growth in mice with subcutaneous xenograft more than in the corresponding groups (Figure S4A). The reduced Ki‐67 expression in xenograft tumours of LY294002‐treated mice also implied that PI3K/AKT signalling inhibition had the ability to counteract the MRPS16 influence on enhancing cell proliferation (Figure S3D). Collectively, MRPS16 might enhance glioma progression via PI3K/AKT signalling activation.

### 3.6 Frataxin is involved in MRPS16‐mediated tumour cell proliferation, migration and invasion

**FIGURE 6:**
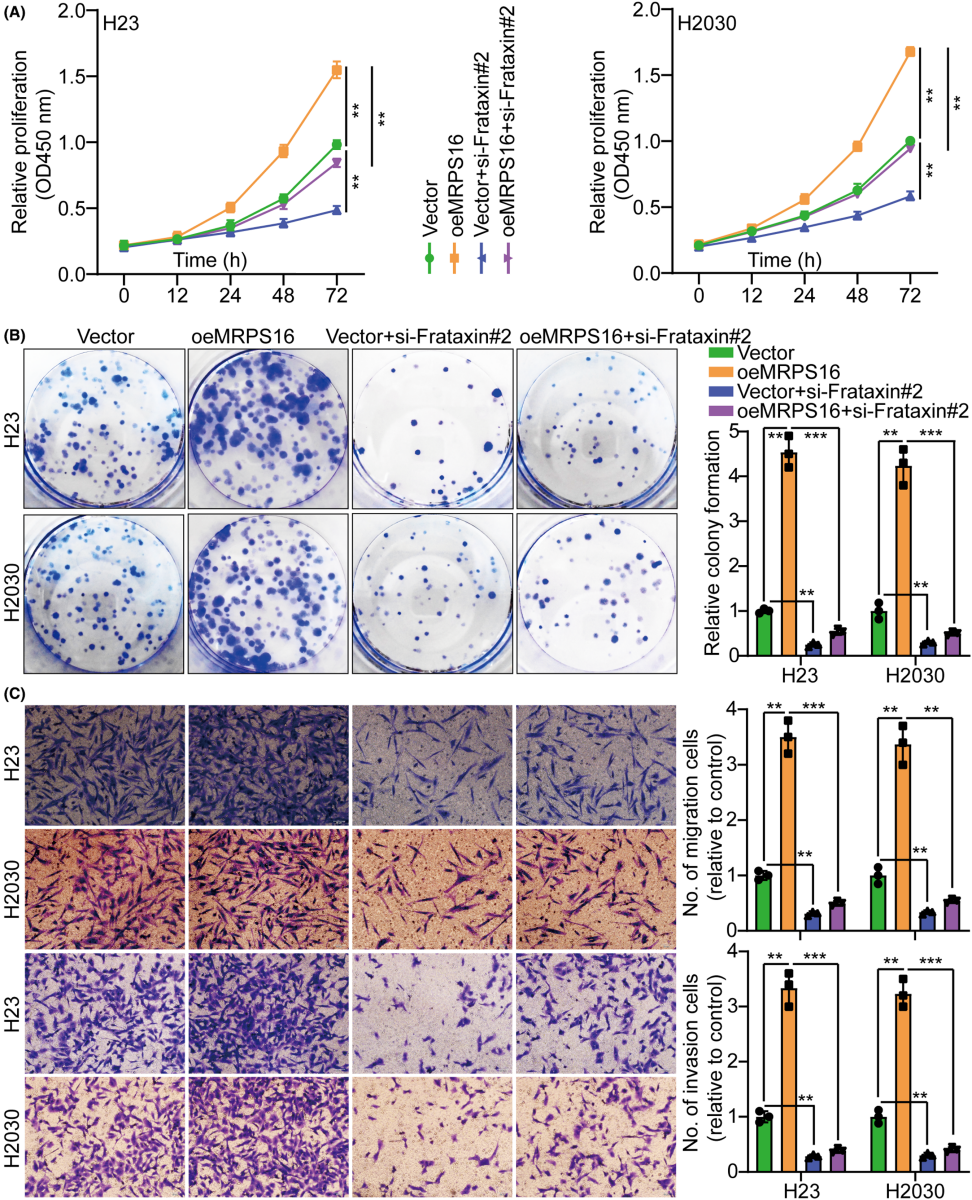
Frataxin is involved in MRPS16‐mediated tumour cell proliferation, migration and invasion. (A,B) MRPS16 overexpression could promote tumour cell proliferation, and the effect could be attenuated by knocking down Frataxin. (C) MRPS16 overexpression could promote tumour cell migration and invasion, and the effect could be attenuated by knocking down Frataxin. The left side represents the histogram. Data were presented as mean ± SD from three independent experiments. **p < 0.01 and ***p < 0.001.

Frataxin is a highly conserved nuclear‐encoded mitochondrial protein that is overexpressed in highly metabolic‐rate tissues, including the heart, neurons, kidney and liver,^23^, ^24^, ^25^ as well as it is involved in various cellular processes and functions in several ways.^26^, ^27^, ^28^ In H23 and H2030 cells, short hairpin RNAs (siRNAs) targeting Frataxin were utilized to investigate further its role in MRPS16‐mediated tumour cell proliferation, migration and invasion. As si‐Frataxin #2 with the highest knockdown efficiency, it will be used as a follow‐up study (Figure S4B). Our previous data showed that Frataxin expression was suppressed by MRPS16 knocking down while promoted by MRPS16 overexpression (Figure 4A). Besides, to verify whether MRPS16 regulates Frataxin expression further through PI3K/AKT pathway activation, MRPS16 was knocked down and overexpressed in H23 and H2030 cells, respectively, revealing that Frataxin was downregulated by MRPS16 knocking down while was promoted by MRPS16 overexpression (Figure 4B). Frataxin silencing attenuated the promoting impacts of MRPS16 overexpression on H23 and H2030 cell proliferation (Figure 6A,B and Figure S5A), migration and invasion (Figure 6C), which was parallel to the in vitro study, where Frataxin silencing attenuated the promoting influences of MRPS16 overexpression on H23 and H2030 cell growth in mice with subcutaneous xenograft more than in the corresponding groups. The reduced Ki‐67 expression in xenograft tumours of Frataxin silencing groups also implied that Frataxin silencing had the capability to counteract the MRPS16 influence on promoting cell growth (Figure S5B). Collectively, MRPS16 enhances tumour development through PI3K/AKT/Frataxin signalling axis activation.

## 4 DISCUSSION

Although MRPS16 dysregulation and its biological role in several disorders have been reported before,^7^, ^8^, ^9^, ^15^ its possible role in LAUD remains unreported.

Herein, the MRPS16 clinical relevance as a prognostic marker as well as its biological role in LAUD was reported, revealing that the overexpressed MRPS16 in human LAUD tissue and cell lines improved LAUD cells growth, showing that MRPS16 is involved in LAUD. Mechanistically, MRPS16 may enhance tumour progression through PI3K/AKT/Frataxin signalling pathway activation.

MRPS16 was reported to function as an oncogene and is involved in the transformation and tumour progression. Wang et al^15^ reported that MRPS16‐OE improves glioma progression through PI3K/AKT signalling activation. Miller et al^7^ indicated that ribosomal protein (MRPS16) mutations lead to defects in mitochondrial translation. Emdadul et al^8^ indicated that mutated mitochondrial ribosomal protein S16 (MRPS16) affects human mitochondrial large and small ribosomal subunit assembly. Nevertheless, in LAUD, neither the function nor the mechanism of MRPS16 was clarified. Herein, MRPS16 was overexpressed in LAUD, and MRPS16 overexpression conferred a poor prognosis and could be an independent prognostic LAUD marker. Based on the loss/gain of function, MRPS16 promoted tumour cell growth. Moreover, Cignal finder cancer 10‐pathway reporter array revealed that PI3K/AKT signalling axis showed a significant inhibition due to MRPS16 knocking down in H23 and H2030 cells, in contrast to the other signalling axis. Additionally, GSEA results revealed that PI3K/AKT signal path might be associated with MRPS16 function in the tumour, which was validated further with WB and rescue experiments by the widely used PI3K/AKT inhibitor LY294002.^29^, ^30^, ^31^ The PI3K/AKT signal path, which is one of the most active pathways in tumours, was reported to be involved in malignancies. Wei C et al^32^ reported that by upregulating PI3K/AKT/MYC signal path, LPCAT1 promotes LAUD brain metastasis. Liang et al^33^ reported that Mex3a interacts with LAMA2 to enhance LAUD metastasis via PI3K/AKT pathway. Gao et al^34^ reported that lung cancer inhibition by IFN‐γ relates to PD‐L1 expression and is governed by PI3K‐AKT signalling. Chen et al^35^ reported that in LAUD, CCAT1/FABP5 promotes tumour progression by mediating fatty acid metabolism and stabilizing PI3K/AKT/mTOR signalling. The above‐mentioned reports, besides our work, indicate that this PI3K/AKT signal path enhances malignant progression in tumours. The Frataxin protein participates in various cellular processes and functions in various ways.^24^, ^36^, ^37^ Interestingly, previous research also showed that Frataxin could promote glioma growth.^16^, ^28^, ^38^

By reviewing the literature, we have currently retrieved only 10 articles related to MRPS16 on Pubmed, so there are relatively few related studies on MRPS16. Wu et al^15^ reported that the facilitation of tumour progression is attributed to MRPS16 through the activation of the PI3K/AKT/Snail signalling axis. Mager et al^10^ reported that analysis of gene expression in mammalian mitochondrial ribosomal proteins. Elpeleg et al^7^ reported that the occurrence of a ribosomal protein (MRPS16) mutation leads to impaired mitochondrial translation. Zhu et al^39^ reported that MRPL15 represents a newly identified prognostic biomarker and potential therapeutic target in the context of epithelial ovarian cancer. Saada et al^8^ reported that this study investigates the impact of mutated mitochondrial ribosomal proteins S16 and S22 on the assembly process of the small and large ribosomal subunits within human mitochondria.

Our results also support that Frataxin could promote LAUD growth. Our data not only indicated that MRPS16 activated the PI3K/AKT/Frataxin axis to facilitate tumour cell proliferation, migration and invasion but also that MRPS16 activated PI3K/AKT signalling, which promoted Frataxin protein expression and promoted LAUD progression. In conclusion, Frataxin is regulated by MRPS16 through PI3K/AKT pathway activation, and our results provide a model via which MRPS16 accelerates LAUD progression. As a result, we suggest and elucidate that MRPS16 enhances LAUD progression via the PI3K/AKT/Frataxin signal path and that these molecules may be promising novel targets.

## AUTHOR CONTRIBUTIONS

**Zai xing Cheng:** Conceptualization (equal); data curation (lead); formal analysis (lead); funding acquisition (equal); investigation (equal); methodology (equal); project administration (equal); resources (equal); software (equal); supervision (equal); validation (equal); visualization (equal); writing – original draft (lead); writing – review and editing (equal). **Ka ming Xue:** Conceptualization (supporting); data curation (supporting); investigation (supporting); methodology (supporting); resources (supporting); software (supporting); visualization (supporting). **Cui Xiong:** Data curation (supporting); formal analysis (supporting); investigation (supporting); methodology (supporting); resources (supporting). **Zhi kun Zheng:** Data curation (supporting); formal analysis (supporting); methodology (supporting); resources (supporting); validation (supporting). **Jin song Li:** Conceptualization (supporting); data curation (supporting); formal analysis (supporting); investigation (supporting); methodology (supporting); resources (supporting); supervision (supporting); validation (supporting). **xin wei Qiao:** Conceptualization (equal); data curation (equal); formal analysis (equal); funding acquisition (equal); investigation (equal); methodology (supporting); project administration (lead); resources (equal); software (supporting); supervision (supporting); validation (supporting); visualization (equal); writing – original draft (equal); writing – review and editing (lead).

## CONFLICT OF INTEREST STATEMENT

The authors declare that they have no competing interests.

## Supporting information


Appendix S1.

## Data Availability

The data sets used during the present study are available from the corresponding author upon reasonable request.
