# Supplementary material for: MRPS16 promotes lung adenocarcinoma growth via the PI3K/AKT/Frataxin signalling axis
Source: J Cell Mol Med. 2024 Mar 20;28(7):e18166. doi: 10.1111/jcmm.18166 (PMC10951875; doi:10.1111/jcmm.18166)
Supplement: Supplementary file 1 — Appendix S1. [file JCMM-28-e18166-s001.docx]

**MRPS16 promotes Lung adenocarcinoma growth via the PI3K/AKT/Frataxin signaling axis**

Zaixing Cheng^1^, Kaming Xue^2^, Cui Xiong^3^, Zhikun Zheng^1^, Jinsong Li^1^, Xinwei Qiao^1^

**Materials and Methods**

**Reagents and cell lines**

Normal lung epithelium MEL12 and NL20, multiple LAUD cell lines H1755, H23, H2030, and H1734 were cultivated according to the supplier’s cultivation suggestion. LY294002 (2-morpholino-8-phenyl-4H-chromen-4-one) was procured from Sigma-Aldrich Co. (St. Louis, MO). Primary antibodies were utilized for detecting MRPS16 (ab151693; Abcam), AKT (ab179463; Abcam), p-AKT (ab192623; Abcam), PI3K (ab191606; Abcam), and p-PI3K (ab182651; Abcam).

**Plasmids, small interfering RNA (siRNA), and transfection**

The H23 and H2030 cells were transfected with the shRNAs targeting MRPS16, and Frataxin using Lipofectamine 3000 (Invitrogen). We constructed the MRPS16 pcDNA3.1 (MRPS16-OE (overexpression)) inserting the respective genes into the pcDNA3.1 vector (Invitrogen). Empty pcDNA3.1 vector (Vector) was used as control. All the siRNAs and shMRPS16/Frataxin and shNC lentiviral vectors were obtained from GeneChem Co., Ltd (Shanghai, China). Supplementary Table 3 lists the sequences of relative siRNAs and shRNAs.

**Real-time quantitative RT-PCR (qRT-PCR)**

Total RNA was isolated from the cells and exosomes using TRIzol and TRIzol LS reagents (Life Technologies). The miRNAs were reverse transcribed using the Mir-X™ miRNA First-Strand Synthesis Kit (Clontech, Mountain View, CA, USA). Real-time PCR was performed using the SYBR Green PCR Master Mix (Takara, Shiga, Japan) and the primers listed in Supplementary Table 4. The mRNA levels were measured with the 7500 Fast Real-Time PCR Systems (Applied Biosystems, Foster City, CA, USA). β-actin was used as internal control.

**Colony formation assay**

Four hundred cancer cells were seeded in a 6-cm plate using two ml of complete medium. Over a period of two or three weeks, the medium was changed twice a week at 37°C with 5% CO2 . Colonies were then washed twice with PBS and fixed with paraformaldehyde at 4%. Colonies were stained with crystal violet (Servicebio, Hubei, China). Counting colonies under an inverted microscope was performed at the end of the experiment. At least three independent experiments were conducted.

**Cignal finder cancer 10-pathway reporter array**

All operations were performed according to the reagent manufacturer's instructions. Cells were resuspended and plated into 96-well plates along with luciferase reporters targeted at common cancer pathways. Luciferase activity was then detected after the cells were incubated.

**EdU proliferation assay**

H23 and H2030 cells were stained with EdU fluorescence following the indicated treatment according to the manufacturer's instructions (Click-iT^®^ EdU Imaging Kits, Invitro). After being cultured in a 24-well plate at 30,000 cells per well, EdU-labeled cells were incubated with 3.7% formaldehyde for an additional 2 h before being fixed at room temperature. After removing the fixative, the cells in each well were washed twice with PBS containing 3% BSA. After removing the BSA, each well was incubated for 20 minutes at room temperature with 0.5% Triton® X-100 in PBS (Sigma, San Francisco, CA, USA). Each well was washed twice with 3% BSA in PBS before being reacted with 500 mL of one Click-iT® reaction cocktail for 30 minutes at room temperature. Then, 1 ml of Hoechst 33342 solution (Sigma, San Francisco, CA, USA) was added to each well for 30 minutes at room temperature. After Hoechst 33342 solution was removed, EdU-labeled cells were counted using fluorescence microscopy and normalized to Hoechst-stained cells. A percentage of EdU-positive (EdU+) cells was calculated using Image-Pro Plus software (Version 6.0, Media Cybernetics, Bethesda, MD, USA).

**Tumor xenograft model**

All male nude mice were obtained from Beijing Vital River Animal Technology Co. Ltd. (Beijing, China). To investigate MRPS16's in vitro growth effect , experimental groups consisted of three or five animals , H23 and H2030 cells (3 × 10^5^) expressing control shRNA or MRPS16 shRNA in 100μl PBS were injected into the subcutaneous tissue of the nude mice. After 5 weeks of injections, tumors were isolated, photographed, and weighed. The Tongji Medical College Ethics Committee approved all experimental procedures .

**Bioinformatic analysis**

All datasets were downloaded from The Cancer Genome Atlas (TCGA) **(**<https://cancergenome.nih.gov/>**)** and Genotype Tissue Expression (GTEx) database. UCSC XENA (https://xenabrowser.net/datapages/) RNAseq data in TPM format of TCGA and GTEx uniformly processed by the Toil process. RNAseq data in TPM (transcripts per million reads) format and log2 transformation for expression comparison between samples. RNAseq data in FPKM (Fregments Per Kilobase per Million) format was converted into TPM (transcripts per million reads) format and log2 conversion was performe. Prognostic data from the following related articles. The results of the independent sample *t*-tests comparing the two groups are expressed as *t*-test values.

**Supplementary Tables 1:** Correlation of the expression levels of MRPS16 in LAUD tissues with clinicopathologic features.

| **Characteristics** | **No. of cases (%)** | **MRPS16** | | **P-value** |
| --- | --- | --- | --- | --- |
|  |  | **Low** | **High** |  |
| **Age (years)** |  | | | |
| ＜50 | 35 (35.7%) | 12 | 23 | 0.2 |
| ≥50 | 63 (64.3%) | 30 | 33 |  |
| **Gender** |  | | | |
| Male | 58 (59.2%) | 24 | 34 | 0.71 |
| Female | 40 (40.8%) | 18 | 22 |  |
| **Tumor size (cm)** |  | | | |
| ＜3 | 33 (33.7%) | 7 | 26 | 0.002** |
| ≥3 | 65 (66.3%) | 35 | 30 |  |
| **Tumor location** |  | | | |
| left | 45 (45.9%) | 18 | 27 | 0.59 |
| right | 53 (54.1%) | 24 | 29 |  |
| **Distant metastasis** |  | | | |
| M0 | 93 (94.9%) | 42 | 51 | 0.05 |
| M1 | 5 (5.1%) | 0 | 5 |  |
| **TNM stage** |  | | | |
| I | 23 (23.5%) | 10 | 13 | 0.002** |
| II | 37 (37.8%) | 25 | 12 |  |
| III | 35 (35.7%) | 7 | 28 |  |
| IV | 3 (3.0%) | 0 | 3 |  |
| **T stage** |  | | | |
| T1 | 30 (30.6%) | 22 | 8 | 0.0005*** |
| T2 | 42 (42.9%) | 18 | 24 |  |
| T3 | 20 (20.4%) | 2 | 18 |  |
| T4 | 6 (6.1%) | 0 | 6 |  |
| **Lymph node metastasis** |  | | | |
| N0 | 50 (51.0%) | 35 | 15 | p<0.0001 |
| N1 | 18 (18.4%) | 4 | 14 |  |
| N2 | 26 (26.5%) | 3 | 23 |  |
| N3 | 4 (4.1%) | 0 | 4 |  |
| **Mortality** |  | | | |
| Survive | 45 (45.9%) | 18 | 27 | 0.01* |
| die | 53 (54.1%) | 24 | 29 |  |

statistics based on available data. *<0.05, **<0.01 and ***<0.001. MRPS16 high expression: score 8-16; low expression: score 0-7.

**Supplementary Tables 2:** Univariate and multivariate for clinicopathological features associated with various prognostic parameters of 98 LAUD patients by Cox-regression analysis.

| Variables | Univariate analysis | | Multivariate analysis | |
| --- | --- | --- | --- | --- |
|  | HR (95%CI) | P-value | HR (95%CI) | P-value |
| TNM stage (I+II vs III+IV) | 1.596 (1.275-2.968) | 0.012* | 1.738 (1.419-3.154) | 0.026* |
| Tumor size (≥3cm vs ＜3cm) | 1.437  (1.246-3.241) | 0.009** | 1.375 (1.128-3.145) | 0.005** |
| T stage (T1+T2 vs T3+T4) | 2.428 (1.368-3.857) | 0.0038** | 2.694 (1.586-3.965) | 0.0025** |
| Lymph node metastasis (Yes vs No) | 3.562 (1.694-5.462) | p<0.0001*** | 2.864 (1.275-3.863) | 0.0009*** |
| Mortality (Survive vs die) | 1.032 (0.736-2.685) | 0.035* | 1.117 (0.714-2.973) | 0.043* |
| MRPS16 (Low vs High) | 1.285  (1.056-2.693) | 0.031** | 1.293 (0.965-2.953) | 0.04* |

| **Gene** | **Primer** | **Sequence(5′-3′)** |
| --- | --- | --- |
| si-MRPS16#1 | forward | CCTCCCAAAGTGCTAGGATTA |
| si-MRPS16#1 | forward | GCATCCTATGATGATCACAAA |
| si-Frataxin#1 | forward | GCAGACGCCAAACAAGCAAAT |
| si-Frataxin#2 | forward | GCTGGACTCTTTAGCAGAGTT |

**Supplementary Tables 3:**

**Supplementary Tables 4:**

| **Gene** | **Primer** | **Sequence(5′-3′)** |
| --- | --- | --- |
| MRPS16 | forward | ACTCGTTGCCCTCAACCTAGA |
|  | reverse | GCAGTCTCTCAGCATTTGTGA |
| Frataxin | forward | CTCGCAGACAAGCCCTATACC |
|  | reverse | GCCCAGCTTAATGGTGAGCA |
| β-actin | forward | ATTGCCGACAGGATGCAGAA |
|  | reverse | GCTGATCCACATCTGCTGGAA |

**Supplementary figure legends**

**
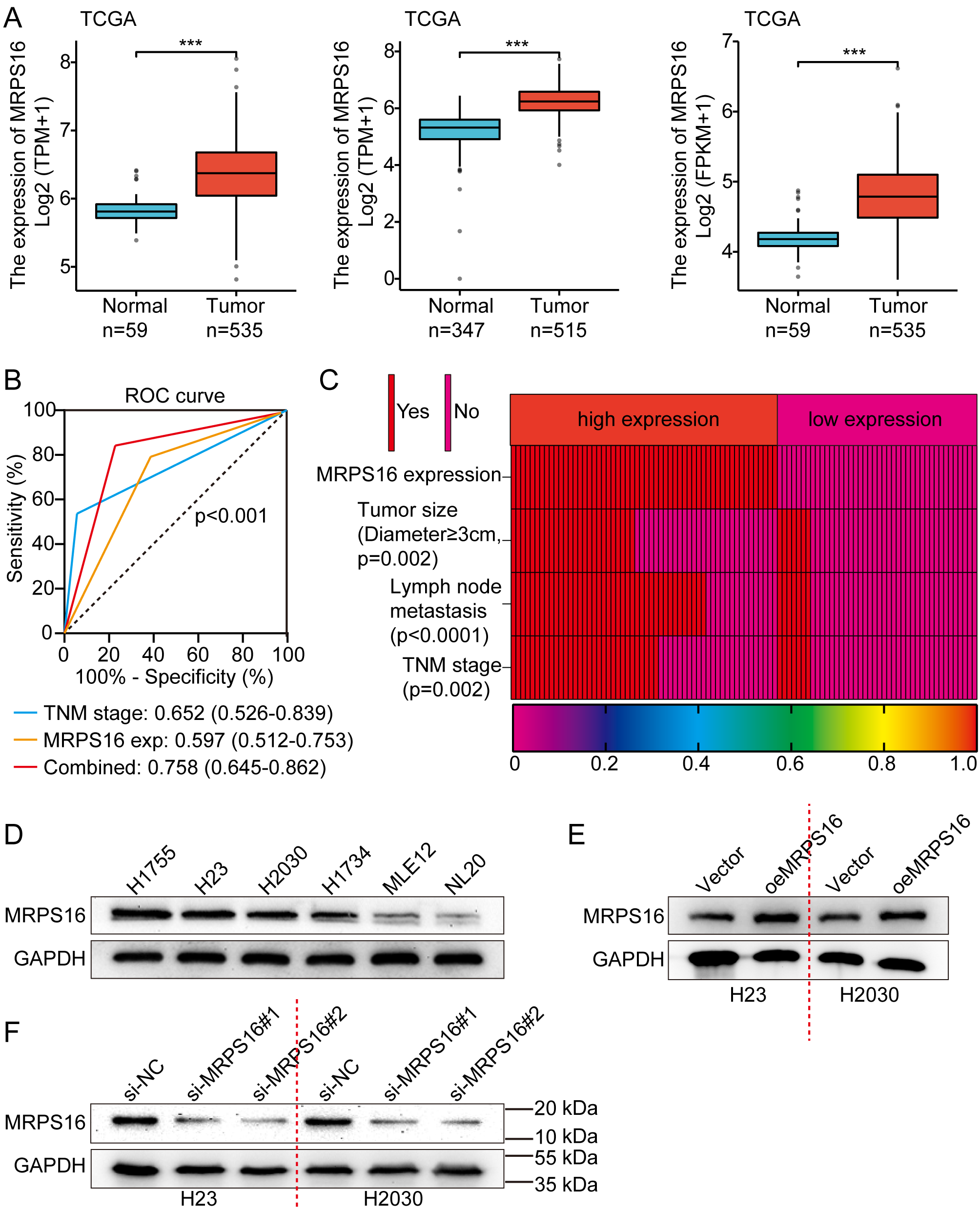
**

**Figure S1 A** The results of the TCGA database showed that MRPS16 was highly expressed in tumor tissues**.** **B** ROC analysis of MRPS16-based, WHO-based and the combination model in predicting clinical outcome. **C** The heatmap illustrates the association of different clinical characters with MRPS16 high and low-expression tumors. **D** WB to detect the expression levels of MRPS16 between MEL12, NL20, H1755, H23, H2030, and H1734 cells. **E-F** Validation of overexpression and knockdown efficiency of MRPS16 by WB. Data shown are mean ± SD (n = 3). (***P < 0.001).

**
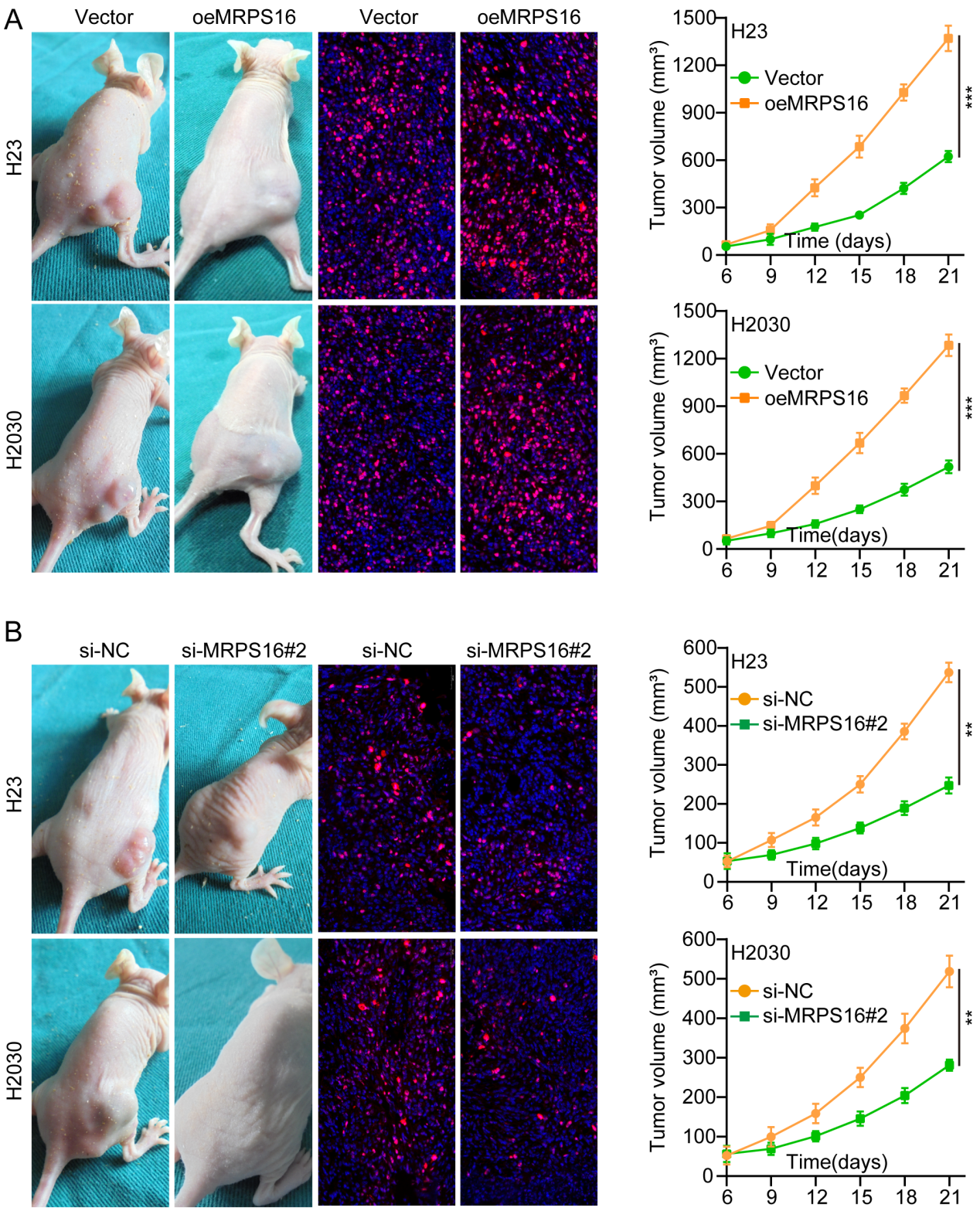
**

**Figure S2 A** Left: Typical pictures of live animal imaging between Vector and oeMRPS16. Representative pictures of Ki-67 staining between Vector and oeMRPS16. Right: Typical animal tumor volume histogram between Vector and oeMRPS16. **B** Left: Typical pictures of live animal imaging between between si-NC and siMRPS16#2. Representative pictures of Ki-67 staining between si-NC and siMRPS16#2. Right: Typical animal tumor volume histogram between si-NC and siMRPS16#2. Data were presented as mean ± s.d from three independent experiments. ** *P* < 0.01 and *** *P* < 0.001.


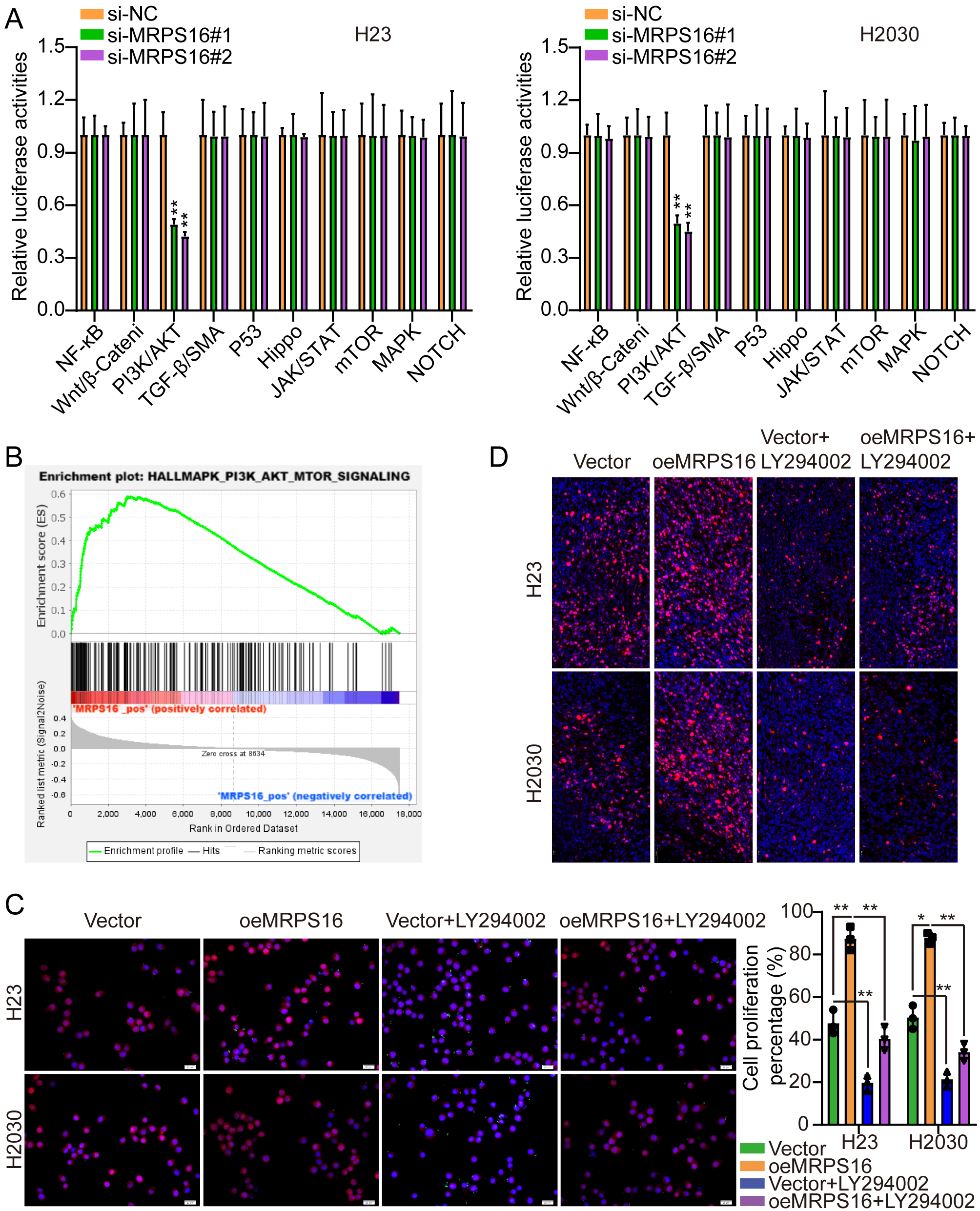


**Figure S3 A** Luciferase reporter gene assay results between si-NC, siMRPS16#1 and siMRPS16#2. **B** GSEA was performed using the GSEA software. **C** Representative images of the EdU between Vector, oeMRPS16, Vector+LY294002 and oeMRPS16+LY294002. Right: Typical histogram Vector, oeMRPS16, Vector+LY294002 and oeMRPS16+LY294002. **D** Typical IF picture and histogram of Ki-67 between Vector, oeMRPS16, Vector+LY294002 and oeMRPS16+LY294002.

The means ± SDs are provided (n=3). *P < 0.05 and **P < 0.01 according to two-tailed Student t tests or one-way ANOVA followed by Dunnett tests for multiple comparisons.


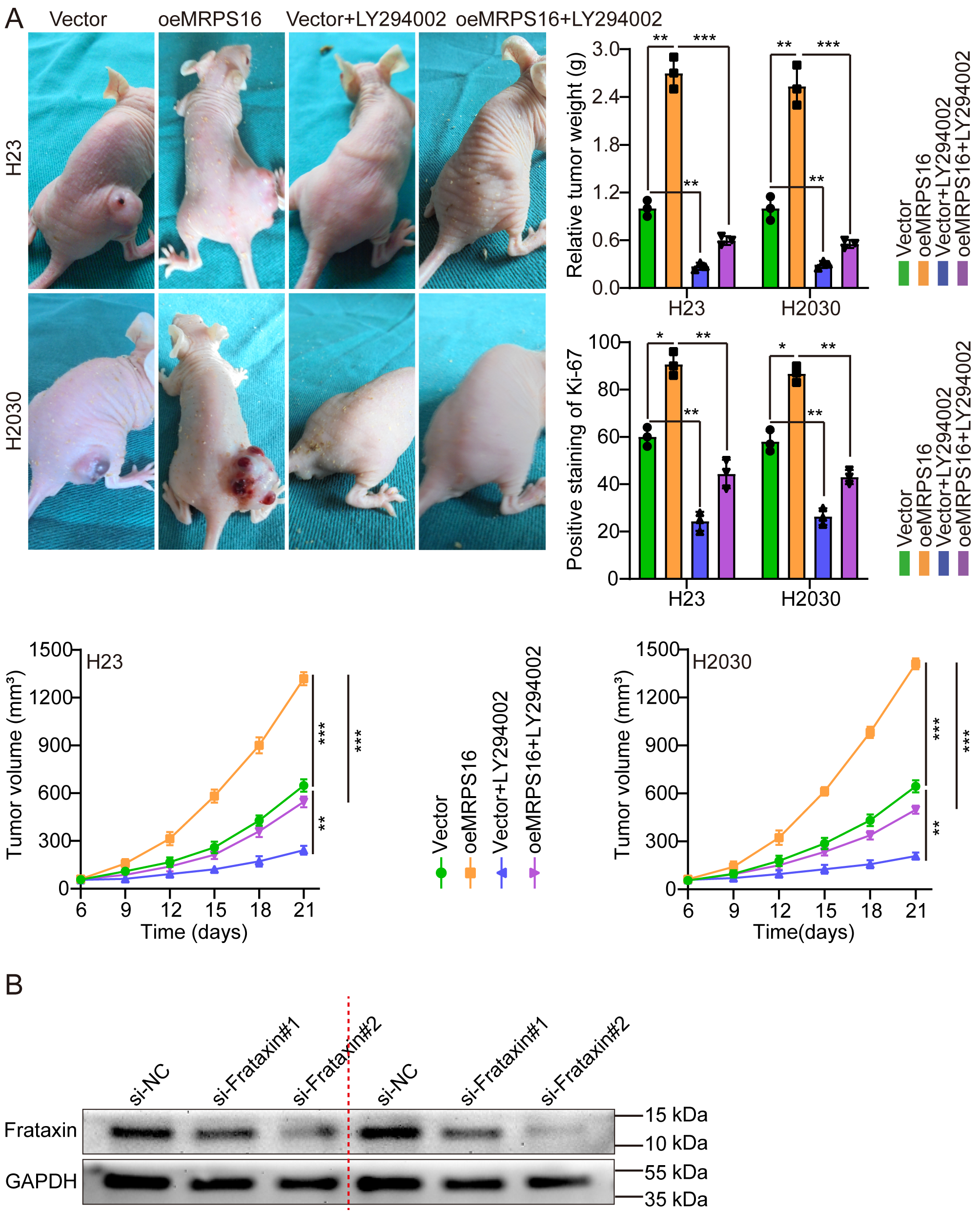


**Figure S4**

**A** Left: Typical pictures of live animal imaging between Vector, oeMRPS16, Vector+LY294002 and oeMRPS16+LY294002. Right: Typical animal tumor weight histogram and histogram of Ki-67. Down: Typical animal tumor volume histogram between Vector, oeMRPS16, Vector+LY294002 and oeMRPS16+LY294002. **B** Validation of knockdown efficiency of Frataxin by WB. The means ± SDs are provided (n=3). *P < 0.05, **P < 0.01 and ***P < 0.001 according to two-tailed Student t tests or one-way ANOVA followed by Dunnett tests for multiple comparisons.


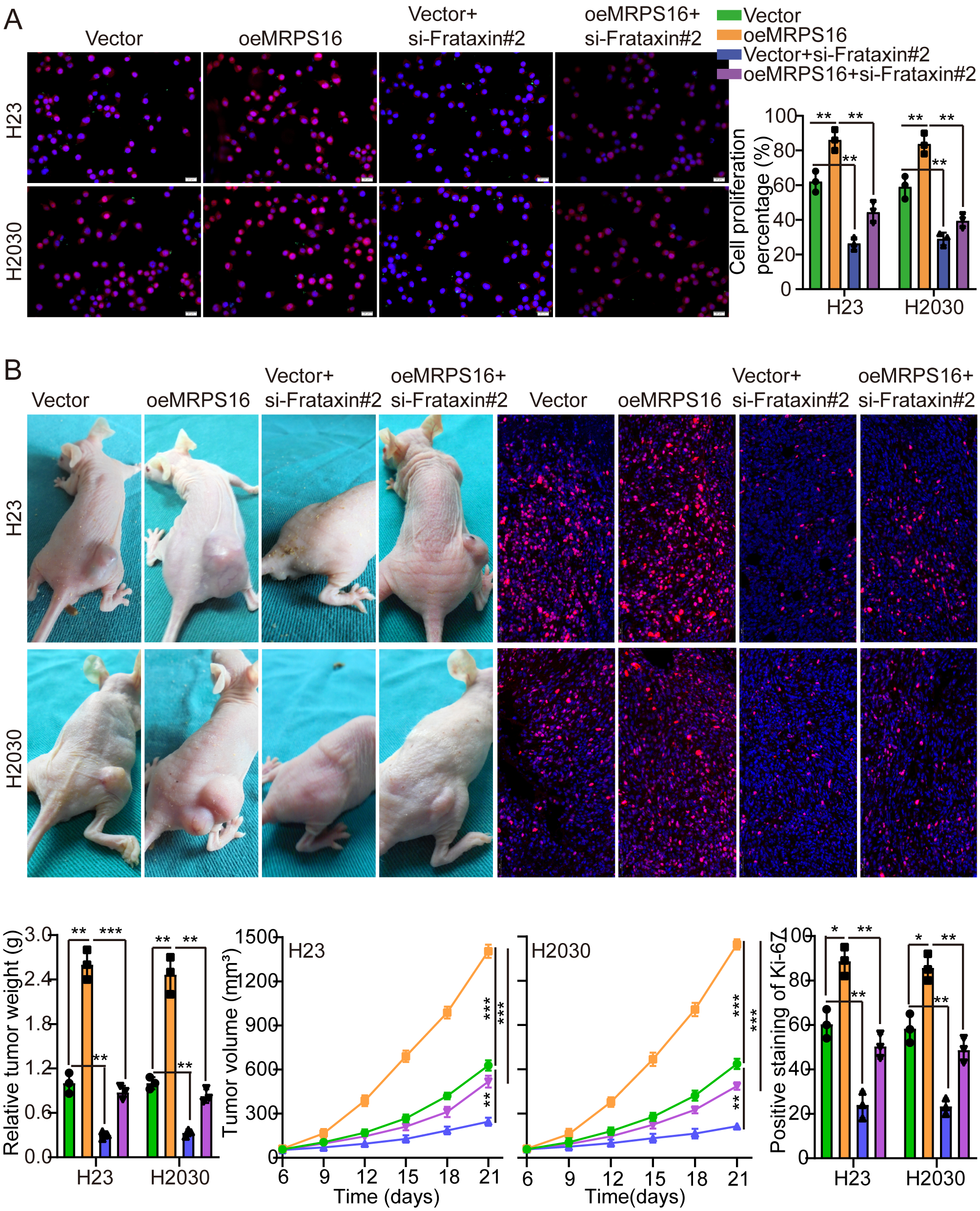
**Figure S5**

**A** Representative images of the EdU between Vector, oeMRPS16, Vector+si-Frataxin#2 and oeMRPS16+si-Frataxin#2. Right: Typical histogram of cell proliferation percentage. **B** Left: Typical pictures of live animal imaging between Vector, oeMRPS16, Vector+si-Frataxin#2 and oeMRPS16+si-Frataxin#2. Right: Representative pictures of Ki-67 staining between Vector, oeMRPS16, Vector+si-Frataxin#2 and oeMRPS16+si-Frataxin#2. Down: Typical animal tumor weight and volume histogram and histogram of Ki-67 between Vector, oeMRPS16, Vector+si-Frataxin#2 and oeMRPS16+si-Frataxin#2. The means ± SDs are provided (n=3). *P < 0.05, **P < 0.01 and ***P < 0.001 according to two-tailed Student t tests or one-way ANOVA followed by Dunnett tests for multiple comparisons.
